# The Political Economy of the Ebola Virus Disease (EVD); Taking Individual and Community Ownership in the Prevention and Control of EVD

**DOI:** 10.3390/healthcare3010036

**Published:** 2015-01-28

**Authors:** Titilola T. Obilade

**Affiliations:** Learning Sciences and Technology, Virginia Polytechnic Institute and State University, 144J Smyth Hall, Blacksburg, VA 24061-0488, USA; E-Mail: obilade@vt.edu

**Keywords:** Ebola virus disease, West Africa, political economy, socio-economic, outbreak, stigmatization, Nigeria, ownership, community, Ebola

## Abstract

The outbreak of an emerging infectious disease of zoonotic origin has exposed the weaknesses in the health systems of the nations affected. The purpose of this paper was to explore the political economy of the existing outcome of the management strategies. In addition, it proposed a new strategy in the management of the current Ebola virus disease (EVD) outbreak. This paper admits that the current management strategy which is a top to bottom approach has not worked in reducing the spread of the disease. Instead of waiting for the disease before treatment is commenced, this paper suggests aggressively preventing infection from the EVD. It presents a bottom to top approach where there is individual ownership and community ownership in the prevention and control of the EVD outbreak. In addition, the paper presents the socio-economic situation of the three most affected countries including the ecology and stigmatization of EVD. It highlights the need for cross border surveillance across the West African nations to prevent importation of the disease as occurred in Nigeria and Senegal. It points out the need for aggressive international cooperation, an aggressive prevention and a sustainable control strategy.

## 1. Introduction

The outbreak of an emerging infectious disease of zoonotic origin has exposed the enormous logistical challenges faced by least developed countries in the ongoing Ebola virus disease (EVD) outbreak. Why is the EVD spreading rapidly? Has the international response been proportionate to the devastating impact of morbidity and mortality of the EVD? Are there socio economic strategies that can help break the cycle? How can we take ownership in the prevention and control of EVD?

This paper examines the political, economic and socio cultural factors in the outbreak of EVD and the exponential mortality and morbidity of infected persons across West African nations. Further, it suggests a new strategy to tackle the EVD. Individuals and communities in the affected countries can take ownership of the prevention and control of EVD. While the EVD is not a pandemic, it has all the capabilities of becoming one if the management of the EVD remains the same. Ebola virus disease is one of the emerging viral diseases listed in the World Health Organisation’s International Health Regulation [1]. It is an epidemic and pandemic prone disease. Ebola virus disease used to be known as Ebola Hemorrhagic Fever [2]. The name Ebola is from the River Ebola in the Democratic Republic of Congo (DRC) [3]. In 1976, two outbreaks of viral hemorrhagic fever occurred simultaneously in southern Sudan and in Zaire (now Democratic Republic of Congo) [4,5]. The causative pathogens were later known as Sudan Ebolavirus and Zaire Ebolavirus [4,5].

In this current outbreak, the countries most affected rank the lowest in the 2013 United Nations Development Programme (UNDP) Human Development Index [6]. Guinea ranked 179 out of 187 countries, Liberia ranked 175 out of 187 countries and Sierra Leone ranked 183 out of 187 countries. Liberia was ahead of only twelve other countries; Mali, Guinea-Bissau, Mozambique, Guinea, Burundi, Burkina Faso, Eritrea, Sierra Leone, Chad, Central African Republic, Democratic Republic of the Congo and Niger. The 12 countries that had lower rankings than Liberia are from Africa and seven of these countries are from West Africa [6].

The possibility of aerosol transmission is still controversial as respiratory spread is suspected but has not been demonstrable in humans [7,8]. However, the Ebola virus is a Biosafety Level (BSL) 4 pathogen [7,9]. Health care workers transporting infected nationals out of the affected countries wear personal protective equipment which can also protect against aerosol transmission. Epidemiologic studies of patients infected with the EVD in Kikwit, Democratic Republic of the Congo demonstrated that the risk of infection was increased when a person was admitted to the hospital, when a person visited someone with fever and bleeding, when a person received an injection in a hospital, attended a funeral, when a person had a physical contact with an ill person, when a person prepared a cadaver for burial and being a health worker [8]. On August 8, 2014, EVD was declared a Public Health Emergency of International Concern by the World Health Organisation (WHO) Director General [10]. The WHO Ebola response team estimates that the case fatality in the current outbreak is 70.8% [10].

In 1976, filoviral hemorrhagic fever occurred simultaneously in southern Sudan and in Zaire (now the Democratic Republic of Congo) [4,5]. This was later to be the first reported outbreak of EVD in Africa. Filoviral hemorrhagic fevers are caused by viruses from the order *Mononegavirales* and the family *filoviridae* [11]*.* Three genera make up the *filoviridae* family; the *Marburgvirus*, the *Ebolavirus* and the *Cuevavirus* [11,12]. The *Marburgvirus* and *Cueevavirus* both have a single species in their genera [2,12]. Ebolavirus genera have five species; *Zaire ebolavirus*, *Sudan ebolavirus*, *Cote d’Ivoire ebolavirus*, *Bundibugyo ebolavirus* and the *Reston ebolavirus* [2,12]. The *Reston ebolavirus* has only been isolated in pigs and monkeys but not in humans [2]. The *Bundibugyo* *ebolavirus* has been responsible for EVD in Uganda and in DRC [2,13]. The *Sudan ebolavirus* species was responsible for the 1976 outbreak in southern Sudan and the *Zaire ebolavirus* species was responsible for the 1976 outbreak in Zaire now DRC [4,5]. The ongoing EVD is caused by the *Zaire ebolavirus* [2]. The *Zaire ebolavirus* has a case fatality ratio of 60%–90% and the *Sudan ebolavirus* has a case fatality ratio of 40%–60% [2]. The *Bundibugyo ebolavirus* has a case fatality ratio of 25% [2]. The *Côte d’Ivoire ebolavirus* was the first EVD to occur in West Africa. It occurred in one person [2,14]. A female ethnologist that was performing necropsy on a chimpanzee from the Tai Forest Park got infected and she fully recovered [2,14]. The *Côte d’Ivoire ebolavirus* is also called the *Tai forest ebolavirus* [14].

The incubation period of EVD is 2–21 days with a mean incubation period of 4–10 days [13]. The clinical manifestations in infections with Ebola virus differ with the species but generally, the onset is abrupt [11,13]. There is fever, myalgia and chills. At the peak of the illness, there could be hemorrhagic manifestations. In the terminal stage of the illness, there is shock from multi-systemic failure. There is usually hypovolemic shock from loss of fluids from diarrhea. During the 1976 outbreak in Kikwit, Zaire (now DRC), one of the causes of spread was the reuse of contaminated needles in an underfunded clinic [3]. Transmission has also occurred through skin contact with an infected person and contact with a cadaver [11].

## 2. The Socio Ecology of EVD

The index case of the present EBV outbreak was reported to have occurred in Guéckédou, Guinea in December 2013 [10,15] but it was not until March 12, 2014 that Médecins Sans Frontières in Guinea was alerted [16]. On March 23, the Ministry of Health of Guinea informed the WHO of the EVD outbreak [17]. Liberia formerly declared an EVD outbreak on March 29 [18]. Sierra Leone notified WHO of its first case of EVD on May 25 [18]. The outbreak was initially graded as a level 2 emergency by the WHO Emergency Management Team [19]. On April 25, WHO thought the situation in Guinea had improved significantly because the dates of onset of the last reported cases in Macenta, Dabola, Kissgougou and Djingaraye prefectures were 24, 25, 26 and 31 days, respectively [20]. The grade level was re-graded to 3 because of the severity and the incident of a traveler infected with EVD traveling to Nigeria [18]. It was not until August 8, 2014 that the EVD was declared a Public Health Emergency of International Concern [16]. The lapses in communicating the EVD show a failure in communication at several levels of authority and allowed the EVD to spread from porous remote borders to densely populated urban areas. It also allowed many transmission chains to be formed thereby increasing the number of EVD cases [16]. Phylogenetic analysis of the Ebolavirus genus from laboratory confirmed cases suggests that the Ebolavirus strain responsible for the current outbreak is different from the Ebolavirus strain responsible for the outbreaks in Democratic Republic of Congo and Gabon [16].

Guéckédou, Guinea lies along the bordering towns of Liberia and Sierra Leone making it easy for the disease to spread to the neighboring towns in Liberia and Sierra Leone. The Guinean Ministry of Health reported 49 EVD cases including 29 deaths on March 22, 2014 [17] and by August 31, 2014, the WHO had reported 3685 probable, confirmed, and suspected cases in West Africa, with 2914 in Sierra Leone and Liberia and 771 in Guinea.

On July 24, 2014 a seventh outbreak of EVD started in DRC. It was the seventh outbreak to occur in DRC since the first outbreak in 1976 [21]. The outbreak in DRC was unrelated to the current outbreak in West Africa [22] On November 22, 2014, WHO declared DRC free from EVD transmission [22]. The past experiences in managing six previous outbreaks played a role in quickly curtailing the July outbreak in DRC. The outbreak caused 66 EVD cases and 49 deaths [22,23].

Twelve of the countries in West Africa are in the list of 48 least developed countries. Table 1 shows the list of countries in West Africa by their populations. It also shows the countries that are included in the list of least developed countries (LDC). Guinea, Liberia and Sierra Leone are among the list of least developed countries [24]. The control of EVD in these countries has been challenging for several reasons.

The current EVD outbreak is the first outbreak in West Africa. A Tai Forest Ebolavirus infection in Côte d’Ivoire occurred in a single individual performing a necropsy on a chimpanzee and the patient recovered [14]. Therefore, it cannot be considered an outbreak as such. Before the outbreak, doctors were used to treating diseases that were endemic in the areas. Diseases like malaria, typhoid fever, cholera are endemic in West African countries. It is also highly probable that in their medical schools, the medical students were taught more about the diseases endemic in the areas and had never seen a case of EVD. Therefore, apart from not being able to include EVD in their differential diagnosis, the doctors have not had any training in infection control especially in Biosafety Level-4 pathogens. Biosafety Level 4 pathogens require the highest level of safety whenever they are going to be handled because of their risk of infectivity, risk of transmission by aerolization and because they can cause life-threatening disease for which there is no vaccine or therapy [7,12]. During the months of March and April 2014, the epidemiological situation in Guinea seemed to be stable because there was no reported case of EVD for a period of 25–31 days in four districts in Guinea [20]. This apparent respite might have made the government overlook contact tracing because relatives might have been keeping their families away from the hospital [20,25].

Geographically, some previous outbreaks that occurred in Sudan, DRC, Uganda, Congo, Kenya, Gabon and Angola occurred in remote regions and after recurrent outbreaks, they have been better at managing subsequent ones [9,11,23]. Outbreaks have also occurred in workers in lead and gold mines [11]. The Ebola virus strain that is responsible for this current outbreak is a variant of the Zaire ebolavirus species and is not the same variant of the ebolavirus responsible for previous outbreaks in DRC and Gabon [2,9]. The implication of this is that it is probable that the ebolavirus was residing in a bat living in the forests of Guinea [9,16]. The suspected animal source of the index case has not been identified [2]. However, in a recent study, researchers found out that the index case in this current outbreak, a two year old child was known to have been playing by a hollow tree that housed bats and that the transmission may not have been through bat meat consumption [26].

The Ebola virus was able to spread through the affected countries because the towns that were initially affected lie along the land borders where development has made human movements fluid which makes contact tracing difficult. In addition, development comes with construction of roads and clearing of bushes which brings the suspected animal reservoirs closer to the human populations. Further, meats from bats and monkeys are eaten by some of the inhabitants of these countries and discouraging the inhabitants in these areas from eating such meats has proved challenging [27].

When clinical manifestations eventually occur, they can be easily misdiagnosed as any of the diseases endemic in West Africa. The wide range of the incubation period of the Ebola virus is between two and 21 days making it possible for an infected person to knowingly or unknowingly infect people in another region where there was no previous EVD. This was the case when a symptomatic Liberian took a commercial flight from Liberia to Nigeria [28]. Ebola virus disease was imported to Nigeria, Mali and Senegal from EVD infected persons traveling from Liberia and Guinea [29,30,31,32]. Nigeria’s index case was from Liberia [30]. Travelers from Guinea were responsible for the cases imported into Mali and Senegal [31,32]. Senegal and Nigeria were declared free of EVD on October 17 and 19, respectively, 42 days after the last known confirmed case tested negative for the EVD virus [29].

Persons can knowingly transport the EVD as was the case of the Liberian traveler that traveled to Nigeria [28]. He was under observation in a hospital in Liberia for possible EVD because his sister had died of Ebola [33]. He discharged himself against medical advice, got a clearance from the Liberian deputy minister of finance and flew from Monrovia, Liberia through Accra, Ghana, to Lomé, Togo, changed airplanes and then flew to Lagos, therefore making it almost impossible for the Nigerian surveillance team to detect that he was coming from Liberia [28,33]. He became acutely ill at the airport and was taken to the hospital. At the hospital, he denied exposure to EVD but the Nigerian doctors suspected EVD when he did not respond to antimalarial treatment [28]. The Ebolavirus disease was laboratory confirmed on the third day of his arrival [30]. The narrative of the Liberian traveler highlights the extreme challenges when persons infected or exposed to EVD deny a history of exposure. Ultimately, from the index case in Nigeria, 20 people contracted EVD and eight of them died giving a case fatality ratio of 40 percent which is lower than in Liberia, Sierra Leone or Guinea. Eleven of the people that contracted EVD were health workers and five of the health workers died [30]. From a total of 894 contacts identified, 18,500 face-to-face visits were conducted by contact tracers [28]. Similarly, the index case imported to Senegal was from a Guinean traveler who traveled in a seven person taxi to Senegal [34]. Prior to traveling from Guinea, the traveler’s brother and sister were being treated for EVD in Guinea [34]. The Guinean traveler also denied any contact with an EVD patient. However, the health personnel in Guinea reported the traveler to the Senegalese authorities which showed a collaborative effort between the two countries. The traveler recovered from the EVD [34].

Researchers have also suggested that changes in climatic conditions could have influenced the outbreak [35,36]. The mean temperatures in Guinea were found to be similar to the mean temperatures in DRC and Gabon, countries that have had several outbreaks of EVD [35]. The mean absolute humidity in Guinea was also found to be similar to the humidity in Sudan and Uganda. Southern Sudan and Uganda have had EVD outbreaks [35]. Research studies also suggest that the index case came from Guinea because of the ecological climate. Guinea Forest region has been largely deforested because of logging and clearing of land for agriculture which makes it easier for the animal host to come in contact with humans [36]. In addition to poverty and poor health infrastructure, the prolonged dry seasons may also increase the risk to humans in contact with the animal reservoir of EVD [36].

Governments riddled with civil wars along areas of natural resources are another contributory factor to the present, desperate outcome in the EVD outbreak. The health system on which the EVD control would have depended did not have a solid foundation. The countries affected lack basic infrastructure that can combat the spread of an infectious disease. The natural resources in the affected nations have not lifted these nations out of poverty. Guinea had the world’s largest undeveloped iron ore in 2008 [37]. Although, it is a member of the least developed countries, it is the world’s main exporter of aluminum ore bauxite and holds half of the world’s bauxite reserves [38]. It has large deposits of gold and diamond [38]. These reserves can only be accessed by mining and because of the suspected implication of bats in the spread of EVD; miners have been reluctant to go to the caves [39] which could further affect the economy of the country.

Despite research, the natural host of the Ebolavirus has not been found although rodents and bats have been suspected [13]. However, for the Marburgvirus, the African fruit bat *Rousettus aegyptiacus* is considered the natural host [9,40]. The method of transmission between the fruit bat and the secondary animal is unknown [9]. Human contact with the fruit bat or secondary hosts like monkeys, apes, chimpanzees and other mammalian species can transmit the infection. In humans, transmission is from human to human [9,13].

The political climate in Guinea, Liberia and Sierra Leone is also another factor in the spread of EVD. Liberia, Sierra Leone and Guinea are recovering from civil wars. These three countries are all in the list of the least developed countries despite having natural resources [24,41,42]. Studies have also shown that civil conflicts usually occur around the Mano River region which straddles Guinea, Sierra Leone and Liberia. These regions have diamond, gold and other natural resources [41,42].

When Nigeria had an imported case of EVD from Nigeria, it was able to mobilize resources in collaboration with international agencies and conducted contact tracing, isolation and quarantine [30]. Despite the forceful contact tracing and isolation, Nigeria still lost eight people to EVD including the Liberian traveler that imported the disease [30]. Nigeria was able to quickly contain the EVD because of a forceful intervention and contact tracing both of which required manpower and material resources [30]. The reproduction number is the number of people that can get infected from the index case before any intervention [43]. When the reproduction number is less than one, it implies that the infection is under control.

Epidemiologists can use the reproduction number to estimate the spread or control of the disease. Recent estimates of the reproduction number of the EVD in Guinea, Sierra Leone and Liberia were 1.51, 2.53 and 1.59, respectively [43]. In Nigeria, the mean reproduction number among secondary cases in Nigeria apart from the index case was 0.4 which implies it was under control [30].

The population of Nigeria is 52.3% of the total population of West African countries and makes it the seventh most populous nation in the world [44]. Table 1 shows the list of the West African countries by their mid-2014 populations and Table 2 shows the mid-2014 populations in Africa by their regions. The combined populations of Guinea, Liberia and Senegal are a fraction of the population of Nigeria and about the population of Lagos, Nigeria where the outbreak occurred [44,45]. The city of Lagos, Nigeria where the index case was located is a bustling city with a myriad of global economic activities involving immense travel by road, air and sea [28,30]. If the outbreak was not quickly curtailed, the consequences would have been monumental. The quick diagnosis, timely intervention, careful contact tracing, effective isolation of infected individuals, interagency collaboration and available resources largely contributed to the containment of the EVD outbreak. The outbreak led to contact tracing of 898 contacts and 18,500 face-to-face visits [28] which could only have been possible because Nigeria had the resources to meet their needs. In addition, the Nigerian media was swift in alerting the country of the EVD outbreak. The countries that have been most affected do not have the resources that Nigeria had. Nigeria, Senegal and all other countries should still be on the alert.

The EVD is fraught with stigmatization of the health workers and the survivors [46]. In a qualitative study of health care workers involved in the care of patients during the EVD outbreaks in Uganda, The Democratic Republic of Congo and Republic of Congo, the findings showed that lack of protective equipment, lack of basic equipment, lack of necessary resources especially at the onset of the outbreak and stigmatization were recurring themes in the countries studied [46]. Anthropological studies conducted in Uganda and DRC during the EVD outbreak showed that the government had not paid salaries in many months, the hospitals were understaffed, the nurses were not given enough materials to protect themselves, they did not have training in infection control and there was not enough medicine to treat common ailments [46]. They had gloves but did not have masks. Masks were later supplied before the end of the outbreak. There was no running water, no electricity and there was no disposable waste system [46].

**Table 1:** Mid-2014 populations (in millions) in West Africa by country.

Country	Mid 2014 population in millions [44]	Mid 2014 population in millions × 100/total West African population (339 Million)
Benin (LDC) [24]	10.3	3
Burkina Faso (LDC) [24]	17.9	5.2
Cape Verde	0.5	0.14
Côte d’Ivoire	20.8	6.13
Gambia (LDC) [24]	1.9	0.56
Ghana	27.0	7.9
Guinea (LDC) [24]	11.6	3.4
Guinea-Bissau (LDC) [24]	1.7	0.5
Liberia (LDC) [24]	4.4	1.29
Mali (LDC) [24]	15.9	4.6
Mauritania (LDC) [24]	4.0	1.1
Niger (LDC) [24]	18.2	5.3
Nigeria	177.5	52.3
Senegal (LDC) [24]	13.9	4.1
Sierra Leone (LDC) [24]	6.3	1.8
Togo (LDC) [24]	7.0	2.0

LDC is member of list of least developed country. [24] and [44] are on the reference list.

**Table 2:** Mid-2014 populations (in millions) in Africa.

Regions in Africa	Population mid-2014 (in millions)	Population mid-2014 (in millions) × 100/(total African population (1136)
Africa	1136	100
Sub-Saharan Africa	920	80.9
Northern Africa	217	19.1
Western Africa	339	29.8
Eastern Africa	378	33.3
Middle Africa	142	12.5
Southern Africa	61	5

The Ebola virus disease is spread by a virus but the political economies existing in the countries, the resultant fractured health systems invigorated the spread of EVD in the affected countries. The delay in determining the outbreak to be a Public Health Emergency of International Concern also contributed to the spread. Deeply entrenched cultural burial practices, stigmatization of EVD, dietary practices, mistrust of the government, poor infrastructure, underfunded health systems, civil wars around areas of natural resources impoverishing the people and enormous logistical challenges have contributed to the spread of EVD.

Health care workers attending to EVD patients wear personal protective equipment. The people being treated for EVD may find the idea of being treated by someone in personal protective equipment intimidating and confounding. Invariably, they make up reasons to explain what they think and some of the beliefs lead to distrust of foreign help and distrust of the government. In an anthropological study, the rumor was that the international organizations concocted the EVD [46]. Relatives have forcefully removed EVD patients from hospital thereby increasing the spread [47]. In addition, because EVD can be transmitted by human contact, when some of these patients die, they die alone without any human touch (only gloved hands) which can be very difficult. This is another reason that they do not want to go to the hospital. An anthropological study conducted in Uganda during an EVD outbreak showed that people were afraid of going to the hospital because they feared that they would never see their families again. There was also the “fear of Euro-Americans buying and selling body parts” which became more prevalent when cadavers were put in body bags and buried in airfields without notifying the relatives [48].

## 3. Taking Ownership in the Prevention and Control of EVD

The present management of EVD in the three most affected countries has been a top to bottom approach whereby the person gets sick and is then treated. Sometimes, the person gets sick and out of fear, s/he is kept away from the hospital by family members thereby infecting others. Further, an infected person may die and be left alone out of fear until a health team is notified or until the health team stumbles upon the corpse on the street. The present approach that is being used has not succeeded in reducing the number of infected people. Therefore, we cannot continue with the same strategy.

We need to change our strategy in the management of the EVD. So far, we have been waiting for the people to become sick and then treat them. This approach has not reduced the number of infected persons. Rather, the infection has been escalating. Instead of waiting for the EVD to attack, and then treat, we need to aggressively prevent EVD. I am proposing a community ownership and individual ownership in the prevention and control of EVD. Rather than wait for the EVD to infect a person, I am proposing that each individual in all the severely hit countries should actively take ownership in the prevention and control of EVD. They should monitor their temperatures twice daily. They should reduce the number of times they step out of their homes on a daily basis because each time they step out of the house, they risk getting infected and if they are already infected, they risk infecting others. They should clean and wipe down their house every day including the walls with soap and water. They should remove their shoes before entering their houses. The door knobs and door handles should be wiped at least twice daily with soap and water. Clothes should be washed on the same day they are worn. Clothes and under clothes worn once should not be worn again without washing. Long hair should be packed under a scarf. Long fingernails and toenails should be trimmed short. Public gatherings of any type should be eliminated during these times.

Schools should be used as an avenue to emphasize personal and hand hygiene. Non-touch greeting techniques should be taught in schools and in communities. Schools should not be closed because education is a good resource especially in times of outbreaks like these. School closures should only be considered in densely populated areas where they have access to distant education and only for short periods. Teachers should be trained on the etiology of EVD transmission and then used as a resource for debunking the myths around the EVD as well as teaching personal hygiene. Habits that seem insignificant like quickly borrowing one’s pen to write should be discouraged because the Ebola virus can be spread by contact. Similarly, the sharing of handsets and other commonly shared items should be avoided.

Since markets cannot be closed indefinitely, the market can be open for fewer hours. The money handled in markets should not be kept in parts of the body where it can make contact with sweat. The Ebola virus can be transmitted through body fluids including sweat. The paper money can be disinfected. There should be a daily market cleaning.

**Village Health Workers (VHW’s):** Village health workers are part of the community and live in the community. Village health workers have been used in many rural areas and in remote areas in various parts of the world [49]. They are also called community health workers. Village health workers should be trained and be part of the community so that they feel ownership. They can be trained in basic health care and coordinate activities when a member of the community becomes ill.

**Health Education:** Before the people can take ownership of the control and prevention of EVD they must be convinced that they need to take ownership. They must be convinced of the epidemiology of the disease. A massive dose of health education is necessary in order to convince the people before they can take ownership. All methods of communicating should be channeled through one source to avoid mixed messages. Mobile phones, radios, televisions, posters, newspapers, bill boards, radio jingles and social media should be used for communicating and respectfully debunking the myths surrounding EVD.

**Information Channeling:** At times like these, it is crucial that the messages coming are from one source so as to control the flow of information and to reduce the chances of wrongful interpretation of those messages. The news on EVD should be narrated from a compassionate perspective and rationality and not paranoia.

**Contact Tracing:** If the people have taken ownership of the EVD, they would report incidence of fevers. When ownership of the EVD has taken place, the infected person should self-identify and inform the community who then informs the health team so that they can begin aggressive contact tracing of the possible contacts of the infected person. However, in situations where the most affected countries are so overwhelmed, contact tracing may not be a feasible option but it can be possible with enough human resources and material resources. The incident of the index case in Nigeria prompted face-to-face visits with 18,500 contacts which required huge manpower and other resources which highlight the resources needed in the affected countries [30]. The reproduction number of EVD is 1.51, 2.53 and 1.59 for Guinea, Sierra Leone and Liberia, respectively, which might require even more manpower than Nigeria’s experience [43].

**Isolation:** As part of taking ownership of the prevention and control of the EVD, the people should self-monitor their temperatures daily even if they are not aware that they have been in contact with an infected person. The government should provide thermometers. The community in cooperation with the government can install temperature stations where their temperatures can be checked. These strategies are aggressive and preventive but it is necessary and economical in order to combat a biosafety level 4 pathogen. If they have been in contact with a suspected or confirmed case of EVD, they should self- isolate. Isolation requires a lot of support because it means the person cannot go out to make a living. If the person does not live alone, it means separating himself/herself from the rest of the family and using separate utensils and bed sheets. This is where the community needs to take ownership while the person is isolated. They need to provide meals and other basic necessities while the individual is isolated.

**Training and re-training of health workers and community leaders:** There must be a constant training and retraining of health workers and community leaders on infection control. In the prevailing situations, the fabric of the country is being threatened; the Armed Forces should assist.

**Replacement of lost personnel:** Several health personnel have been lost during this epidemic and they need to be replaced. Volunteers are needed. It is better to have more volunteers than to have insufficient numbers.

**Collaboration with countries that have had several outbreaks of EVD:** West African nations should collaborate with countries that have had repeated outbreaks of EVD. The DRC had its seventh EVD outbreak in July 2014, and by November 2014, it was declared free of EVD transmission [23]. They should also collaborate with countries like Nigeria and Senegal that have successfully stopped the spread of EVD transmission in their countries.

**Build infrastructure:** The UNDP Human Development Index ranking for the three most affected countries ranges between 175 and 183 out of 187 countries and are not in a position to build this infrastructure themselves and need international help.

**International cooperation:** These countries are struggling and need a lot of aggressive international support and cooperation in human and material resources for all the steps outlined under individual and community ownership. 

**Isolation stations:** Isolation stations for EVD patients should be built within a transportable distance so as to encourage people to self-identify and to encourage the community to identify suspected EVD cases. It would also encourage relatives to identify family members that have suspected EVD. If the isolation centers are far from their homes, people would be reluctant to self-identify. If isolation centers cannot be built within a transportable distance, the government might subsidize transportation fares for relatives that have patients in isolation units.

**Cross border surveillance:** Although Nigeria and Senegal have been cleared of EVD transmission, all neighboring countries including the two countries that have been cleared of EVD transmission should continue aggressive cross border surveillance. The index cases in Nigeria and Senegal have proved that people would knowingly deny contact with an EVD patient thereby putting many others at risk. This is a major reason why cross border surveillance must be aggressive and collaborative. The Guinean authorities were able to alert the Senegalese authorities of a Guinean traveler with suspected EVD who had traveled to Senegal showing an effective collaboration because the Guinean traveler was later confirmed to have EVD [34].

**Community surveillance:** Community surveillance is the community making efforts to identify, isolate and report suspected cases.

**Discharge package:** When EVD patients are discharged back to the community, they should be given discharge packages containing basic necessities like mattresses, blankets and beverages as a welcome back gesture because it is to be assumed that, for instance, their mattresses would have been disposed of when they got ill.

**Basic protocol training:** The communities should face the reality of EVD. The outbreak cannot be resolved in a short time and it may not be possible to prevent future outbreaks once the first outbreak is over as several countries in Africa have had repeated outbreaks with a respite of a few years in between outbreaks. However, it is possible to reduce the chances of an outbreak. There should be a basic protocol training of what the individual should expect if they should have EVD. The importance of the health worker wearing personal protective equipment should be explained to them so that they do not become fearful if they get admitted and they see health workers in personal protective equipment. There should also be basic protocol training on what to do and how to care for an individual with suspected EVD before health workers arrive.

**People with special needs:** People with special needs like the visually impaired who are blind and depend on touch to move around should also wash their hands frequently and reduce the number of times they go out. Walking sticks and wheel chairs should be cleaned daily. Those that assist them should ensure that they practice good personal hygiene.

**Provision of water, sanitary toilets and safe refuse disposal:** The government should provide water pumps in areas that do not have water. Sanitary toilets and safe refuse disposal should also be provided because the people cannot safely practice proper personal hygiene if some of their basic needs are not met.

**Land, air and sea ports:** The closure of land, air and sea ports should not be instituted because it could drive more EVD cases underground and increase the spread. The Health Assembly of the World Health Organization can impose sanitary and quarantine requirements if necessary [1].

**Health care workers:** The morale of the health care workers in the affected countries has become low because of a number of reasons including a massive loss of their colleagues. In addition, they are stigmatized. The health care workers in the affected countries need to be counselled by specially trained health care counselors.

## 4. Conclusions

In conclusion, this paper has attempted to describe the impact of EVD disease and suggested a change in the present strategy. There is a need for individual and community ownership in the prevention and control of the disease. The three most affected countries were already having chronic multi-faceted challenges in their health care systems before the disease outbreak. The EVD outbreak only exposed the fragile, fractured health systems in these countries crushing the health systems even further. If it was not EVD, a different infectious disease that spreads rapidly would have exposed the fragility of the health systems. The history of civil wars around the areas of natural resources has not helped the governments of these nations. The international organizations and other countries need to aggressively pursue prevention and control strategies by arming the people with the tools for individual and community ownership. The economic cost of prevention is less than the economic cost of treatment.
